# Acknowledgement to Reviewers of *Pharmaceuticals* in 2019

**DOI:** 10.3390/ph13010014

**Published:** 2020-01-14

**Authors:** Pharmaceuticals Editorial Office

**Affiliations:** MDPI, St. Alban-Anlage 66, 4052 Basel, Switzerland

The editorial team greatly appreciates the reviewers who have dedicated their considerable time and expertise to the journal’s rigorous editorial process over the past 12 months, regardless of whether the papers are finally published or not. In 2019, a total of 179 papers were published in the journal, with a median time to first decision of 15 days and a median time from submission to publication of 38 days. The editors would like to express their sincere gratitude to the following reviewers for their generous contribution in 2019:
Abdel-Hakeem, MohamedLis Arias, Manuel J.Adamson, David CoryLiu, HuolongAlaibac, MauroLlinares, José MiguelAlbericio, FernandoLomberget, ThierryAlexander, DenekaLopez, VictorAlexopoulos, AthanassiosLorberboum-Galski, HayaAli, RameezLucarini, SimoneAmanda, LumsdenLuchinat, EnricoAngelova, SilviaLuque, SoniaAngelucci, AdrianoMadine, JillianAntosiewicz, JędrzejMagda, JulesArmijo, Juan FranciscoMalik, DanishArmon, RobertMalvè, MauroAskes, SvenMannello, FerdinandoAvin, VijayMaria Carmela, Bonaccorsi Di PattiAzaïs, HenriMarra, AlbertoBadea Doni, MihaelaMarrocchi, AssuntaBainbridge, JacciMarrs, James A.Bandea, ClaudiuMartin Lawson, AtaBattula, NarendraMartín Palma, M. ElenaBaudoin-Dehoux, CécileMartins, Ana CatarinaBelaidi, Abdel AliMartins, Andre F.Berens, ChristianMaruani, AntoineBernard, Marek K.Marx, Joannes J. M.Bernardini, GiuliaMatei, IoanaBernatchez, Jean A.Matesanz, Ana I.Berzal-Herranz, AlfredoMaurer, AndreasBesson, ThierryMayence, AnnieBier, DirkMayer-Proschel, MargotBlasiak, JanuszMcLemore, Gabrielle LynnBoccafoschi, FrancescaMeabon, James S.Bolhuis, AlbertMedici, SerenellaBorghaei, Ruth C.Meegan, Mary J.Bouckaert, JulieMelman, ArtemBroecker, FelixMerah, OthmaneBustos, Victor H.merlin, didierBuxton, DenisMertens, ChristinaBylund, DanMesslinger, KarlByun, SanguineMichalik, JanCacciari, BarbaraMiederer, MatthiasCallery, Patrick S.Mielgo, JuanCampanella, AlessandroMikropoulos, ChristosCarboni, LuciaMiller, John H.Carraro, MassimoMishra, Jay S.Carvajal, Asia FernandezMitchell, Cassie S.Castagna, AnnalisaMittal, NiteshCastro, Ricardo A.E.Mobbili, GiovannaCauli, OmarMollica, AdrianoCerón-Carrasco, José PedroMori, NoriyukiCerrada, ElenaMukherjee, SudipCertal, Ana CatarinaMuller, MarcChankhamjon, PranatchareeyaMulloy, BarbaraChen, Shuen-EiMuthu Karuppan, Mohan KumarChen, Qiao-HongMyung, WoojaeChen, GangNagy, CorinaCheng, Juei-TangNairz, ManfredChilders, Delma SusanNamasivayam, VigneshwaranChiou, BrianNanjundan, MeeraChowdhury, RatulNarayanan, S. PriyaChristides, TatianaNardecchia, StefaniaChu, XianpingNastasa, CristinaCicenas, JonasNawrot, BarbaraCieslar, GrzegorzNayak, Usha Y.Cinar, BekirNgezahayo, AnacletCiocan, CorinaNguyen, Hanh ThuyCione, ErikaNitulescu, MihaiCirillo, GiuseppeNitulescu, George MihaiClement, CristinaNowak, AdrianaCollin, Rob W.J.Ntie-Kang, FideleCosti, SaraNuzzo, DomenicoCoyne, CodyOkamoto, ShigefumiCozza, GiorgioOkuda-Ashitaka, EmikoCrisponi, GuidoOnnis, ValentinaCrouzier, ThomasOnyango, IsaacCsávás, MagdolnaOyama, EtsukoCzyz, MalgorzataPacios, Luis F.D’Acquarica, IlariaPaiva, SandraDadachova, EkaterinaPalenzuela López, José AntonioDaley, PeterPantopoulos, KostasDaniel, VyoralPapakyriakou, AthanasiosDardonville, ChristophePappas, ChristosDe Brevern, Alexandre G.Parfrey, HelenDe Caro, VivianaParmeggiani, FabioDe Paola, IvanParra, MargaritaDe Simone, AngelaPatrie, Steven MatthewDeb, SubrataPavela, RomanDell'Agli, MarioPavic, ValentinaDenkova, AntoniaPeifer, ChristianDesai, DhimantPeng, TengDey, AdwitiaPerche, FédéricoDianzani, IrmaPereira, BrunoDietrich, AlexanderPereira, CarlaDimova, ElitsaPérez Pérez, MaríaDing, LingPeriyasamy, PalsamyDinh, Dzung H.Petrak, JiriDistel, MartinPiagnerelli, MichaëlDodi, GianinaPilli, NageswaraDuburs, GunarsPlatas-Iglesias, CarlosDuerfeldt, AdamPouli, NicoleDurand, Jean-OlivierPozo Rodríguez, ManuelDuranti, AndreaPruszynski, MarekDuzgunes, NejatPrzybył, Jarosław L.Eadon, Michael T.Puchkova, Ludmila V.Ear, JasonQi, JunpengEhrnhoefer, DagmarR. Santos, Cleydson BrenoEmmy Lacourt, TamaraRadu, Beatrice MihaelaEngewald, WernerRamadori, GiulianoEppard, ElisabethRedmer, TorbenEritja, RamonReid, Christopher W.Escames, GermaineRen, ZhenhuaEspel, EnricRenaudineau, YvesEsworthy, SteveResch, UlrikeFacchiano, AngeloRidge, Perry G.Fakhrullin, Rawil F.Rishi, GautamFalsini, BenedettoRiska, PaulFan, Hueng-ChuenRivella, StefanoFarkas, MichaelRoby, Justin A.Federico, StephanieRodger, AlisonFélix, LuisRodriguez-Rubio, LorenaFigueiral, Maria HelenaRoetto, AntonellaFigueiras, AnaRondon, AurélieFilaretova, LudmilaRose, KlausFornstedt, TorgnyRozalski, MarcinFossey, John S.Ruchala, PiotrFrancesca, SelminRussu, WadeFrazer, David MRyabchikova, Elena I.Froeyen, MatheusSabatier, Jean-MarcFujita, MikakoSaito, YoshiroGabrielson, Nathan P.Saito, RyokoGanguly, SamitSajeva, MaurizioGarcia-Doval, CarmelaSantamaria, RitaGarcía-Garrido, Sergio E.Santos, ClementinaGarello, FrancescaSaule, SimonGini, GiuseppinaSchols, DominiqueGok, OzgulSeca, Ana Maria Loureiro DaGolombick, TerrySeger, RonyGombert, AlexanderSellars, JonathanGómez-Puertas, PaulinoSerwer, PhilipGoněc, TomášSeto, ShigekiGonzález, MartaSharma, ShwetaGraczyk-Jarzynka, AgnieszkaShimada, YasuhitoGraeve, LutzShimizu, ShunichiGrande Tovar, Carlos DavidShirakawa, SeijiGrant, Gregory A.Si, HongweiGreenwood, Michael T.Siddappa, ByrareddyGriffiths, PeterSieniawska, ElwiraGriñán-Ferré, ChristianSigusch, Bernd W.Grosso, GiuseppeSippl, WolfgangGuerra, BarbaraSkiba, MohamedGuinamard, RomainSmith, Robert A.Guirado, RamonSobotta, ŁukaszGütschow, MichaelSokalski, AndrzejHalaban, RuthSong, Im-SookHamano, TadanoriSoural, MiroslavHanif, MuhammadSousa, Sérgio FilipeHans, Chetan P.Spiegler, VerenaHans, GuySprio, Andrea ElioHarris, Norman R.Sresht, VishnuHasan, S. SaifSrivatsan, MalathiHeggermont, WardSt Hill, CatherineHémadi, MiryanaStanovnik, BrankoHepnarova, VendulaStefane, BogdanHerczegh, PálSu, Wen-YuHevey, RachelSu, MengHomma, Miwako KatoSu, Zheng-YuanHrabal, RichardSugimoto, NaotoshiHsin, Kun-YiSun, XuelongHsu, ToddSun, HaoHu, Dong GuiSuzuki-Kerr, HarunaHuang, Yan-JangŚwietlik, DariuszIijima, NoriakiSzaleniec, MaciejIlia, GheorgheTabu, KouichiImlach, Wendy L.Takeda, ShigekiImperatore, RobertaTakeuchi, TomoharuItarte, EmilioTanabe, ShihoriItoh, YoshifumiTartey, SarangIwatsuki, MasatoTaylor, Alison MarieJablonska, EwaTeng, YongJadeja, RavirajsinhTeng, PengJaki, BirgitThapa, Raj KumarJedelská, JarmilaThevenod, FrankJežek, JanTimmerman, PeterJimi, EijiroTing, Jeffrey M.Jose, JoacimTinoco, Arthur D.Juhasz, BelaTitz, AlexanderJuretić, DavorTonelli, MicheleKadriu, BashkimToovey, StephenKaldis, PhilippTortorella, PaoloKallscheuer, NicolaiTrabocchi, AndreaKanamori, TakaoTrammell, Scott A.Kanda, YasunariTreglia, GiorgioKang, Hee EunTricarico, DomenicoKang, CongBaoTuccinardi, TizianoKarnati, Konda ReddyTurkington, RichardKasperkiewicz, PaulinaTurnaturi, RitaKauffman, Kathryn MarieUchida, TakeshiKawakami, KohsakuUmapathy, GaneshKeith, GordonUsai, MariannaKhan, Meraj AlamVaidyanathan, GanesanKiec-Kononowicz, KatarzynaValsesia- Wittmann, SandrineKiesewetter, Dale O.Van den Bergh, HubertKiljunen, SaijaVan Hecke, KristofKim, Hak JoongVan Noorden, RonKimpel, JanineVankayala, RavirajKishimoto, DaigaVarani, LucaKonc, JanezVeenman, LeoKong, WeiliVelikyan, IrinaKopel, PavelVenkatesan, JayachandranKosik, IvanVerhelst, StevenKozempel, JánVerma, PragyaKozlov, SergeyVictor, BrunoKraus, ChristophViola, GiampietroKrejbich-Trotot, PascaleVolcho, KonstantinKrijt, JanWalsby, CharlesKubinova, SarkaWang, QunKumamoto, EiichiWang, ChenguangKumar, SunilWang, ChunlingKumar Jha, MithileshWang, GuankuiKunda, NiteshWang, TaiKuryłowicz, AlinaWeeks, Robert J.Kwon, Tae-HyukWeidlich, TomášLa Mendola, DiegoWeiss, GunterLa Motta, ConcettinaWen, ZhiweiLachowicz, Joanna IzabelaWessel, Hans PeterLai, Yu-HengWijnholds, JanLaity, Peter RWilliams, Noelle S.Lamar, John M.Wilusz, JeffLambertucci, CatiaWinters, BryonyLambrou, George IWong, Ching OnLamers, FemkeWright, CatherineLartigue, LenaicWunderlich, GerdLatacz, GniewomirXiao, HanLatunde-Dada, Gladys OluyemisiXie, LishiLawen, AlfonsYang, InhoLe Borgne, MarcYe, YunpengLe Moigne, VincentYin, JianLechner, JudithYokoyama, YoshihitoLeclerc, FabriceYoung, Howard A.Lee, SangkyuYousefi, Behrooz H.Lee, GihyunYue, XuyiLee, Yeon SunZagórska, AgnieszkaLehner, MałgorzataZaitsev, AlekseyLemercier, GillesZapico, Sara C.Lewkowski, JaroslawZeilinger, CarstenLi, YunfeiZhang, YijunLi, Ruei-NianZhao, XiaoaiLi, YanZhu, JieLi, WenyiZięba, AndrzejLi, LianboZiesenitz, VictoriaLight, Sharee N.Ziyatdinova, GuzelLima, Sofia CostaŻołnowska, BeataLin, Kuo-ShyanŽuvela, PetarLinder, Maria


